# The kidney of the *Nodularia* freshwater mussel has a larger filtration-size and counter-current system with improved water excretion compared with the seawater mussel *Mytilus*

**DOI:** 10.1242/bio.058692

**Published:** 2021-06-08

**Authors:** Eriko Seo, Hidefumi Wakashin, Yoshiteru Seo

**Affiliations:** 1 Department of Marine Ecosystem Dynamics, Division of Marine Life Science, Atmosphere and Ocean Research Institute, The University of Tokyo, Kashiwa 277-8564, Japan; 2 Department of Regulatory Physiology, Dokkyo Medical University School of Medicine, Tochigi 321-0293, Japan; 3 Division of Internal Medicine, Goi Hospital, Ichihara 290-0056, Japan; 4 Faculty of Home Economics, Aichi Gakusen University, Okazaki 444-8520, Japan

**Keywords:** Nephridia, Glomerular filtration, Counter-current, T_1_ relaxation time, Magnetic resonance imaging

## Abstract

Histological studies and magnetic resonance imaging were employed to analyze the kidney structure and function of the freshwater mussel, *Nodularia douglasiae*. The *Nodularia* kidney consists of proximal, intermediate and distal tubules. The epithelia of the renal tubules were composed of a single layer of cuboidal cells. The proximal and distal tubules run in opposite directions underneath the pericardial cavity. Molecular weight cut-off (MWCO) values for the kidney filtration were detected by MR tracer injections: gadolinium-diethylenetriaminepentaacetic acid (GdDTPA) at 0.55 kDa, an oligomer-based contrast agent (CH3-DTPA-Gd) at 2.2 kDa, as well as Gd-DTPA-polylysine at 10, 22, and 110 kDa. The *T*_1w_-MRI intensity and *T*_1_ relaxation rate (*R*_1_) of the pericardial cavity and renal tubules increased with tracers smaller than 10 kDa. The other tracers showed only minimal or no increase. Thus, we concluded that the MWCO of the kidney is 22 kDa, 50 times larger than that for the *Mytilus* living in seawater. Since the *R*_1_ values of the renal tubules were similar to those of the pericardial cavity, the kidney did not concentrate filtrated tracers. The slow decay of the magnetic resonance (MR) tracers from the renal tubules indicated a low filtration rate, suggesting that the counter-current system reabsorbs useful solutes without reabsorption of water. The higher MWCO may be beneficial to maintain the tubular oncotic pressure and allow excretion of excess water. In conclusion, a main renal function of the freshwater mussel is the excretion of water, opposite to that of the seawater mussel and vertebrates, which preserve water.

## Abstract (precis)

**Summary:** The molecular weight cut-off of the freshwater mussel *Nodularia douglasiae* (22 kDa) is larger than the seawater mussel *Mytilus* (0.5 kDa), and *N. douglasiae* has better water excretion with a counter-current system.

## INTRODUCTION

The characteristics of the excretion systems of bivalves have not been well analyzed to the present date (Martin and Harrison, 1966; Bayne, 1976). The main function of the kidney in a terrestrial habitat is conserving water, in contrast to the loss of water to the air or urine. Seawater mussels also face the risk of losing body water, as it can be extracted by the hypertonic seawater (Bayne, 1976). We found that a seawater mussel, *Mytilus galloprovincialis*, can uptake Mn^2+^ ions from seawater, and can concentrate Mn^2+^ in urine by more than ten times (Wakashin et al., 2018). Thus, the *Mytilus* kidney can concentrate waste substances, such as heavy metals, and may reabsorb water to maintain a certain level of body water (Wakashin, et al., 2018, 2019). We also concluded that the molecular weight cut-off (MWCO) for filtration by the auricular wall of the *Mytilus* is around 0.5 kDa, which is almost 1/100 (Guyton and Hall, 2006) that of the MWCO for filtration by the glomerulus of the kidney in vertebrates, such as humans (Wakashin et al., 2019). Thereafter, we received criticism regarding our data concerning the concentration of Mn^2+^ in the urine and the small MWCO, compared with results reported for freshwater mussels such as the *Anodonta cygnea* (Potts, 1954). In freshwater habitats, there is the risk of dilution of body water due to the inflow of water caused by higher osmotic pressure of the body fluid. Therefore, the kidney of the freshwater mollusks has to excrete any excess amount of water to prevent dilution of the body fluid. In addition, the anatomical structure of the exocrine system of the *Anodonta* varies from that found in the seawater mussel, *Mytilus* (Martin and Harrison, 1966; Martin, 1983; Andrews and Jennings, 1993). Therefore, this study was conducted in order to analyze the function of the exocrine system of a freshwater mussel, the *Nodularia douglasiae*, and to compare our results with those reported for the *Anodonta* and *Mytilus*.

Kidney function was measured noninvasively by using a magnetic resonance imaging (MRI) method and magnetic resonance (MR) contrast agents. The MR contrast agents were used as the extracellular tracers to determine the MWCO of the filtration that takes place in the exocrine system of the *Mytilus galloprovincialis* (Wakashin et al., 2019). Therefore, in this text, we called MR tracers not MR contrast reagents. MR tracers are paramagnetic, and accelerate the longitudinal relaxation rate (1/*T*_1_=*R_1_*). Thus, when MR tracers are injected in the hemolymph of the foot, the *R_1_* is increased, depending on the concentration as follows: *R*_1_=*R*_0_+K·C, where *R*_0_, K and C are the intrinsic *R*_1_ of the hemolymph, the relaxivity value of the MR tracer (mM^−1^ s^−1^), and the concentration of the MR tracer (mM), respectively. *T*_1w_-MRI intensity (M(*R*_1_)) with a short echo-time could be written as follows:
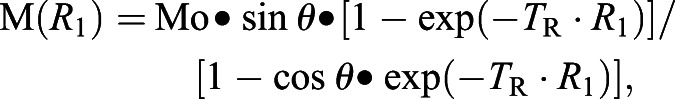
where Mo is the equilibrium image intensity, and *T*_R_ and θ are the repetition time and the flip angle of the excitation pulse, respectively. Thus, an increase in the *R*_1_ could be detected as a higher signal intensity in *T*_1_-weighted MRI (*T*_1w_-MRI) (Wakashin et al., 2018). Therefore, the *R_1_* and *T*_1w_-MRI intensity in the exocrine system should be increased when MR tracers are filtrated and/or concentrated in the lumen.

In order to investigate this hypothesis; (1) we studied the anatomical structure of the exocrine system using histological examinations conducted using a light microscopic method and high-resolution MR imaging, (2) we measured *T*_1w_-MRI and *R_1_* values in the urine in the exocrine system of mussels enhanced by five MR tracers with varying molecular weights from 2.2 to 110 kDa: gadolinium-diethylenetriaminepentaacetic acid (GdDTPA) at 0.55 kDa, an oligomer-based contrast agent (CH3-DTPA-Gd) at 2.2 kDa, as well as Gd-DTPA-polylysine (Gd-PL) at 10, 22, and 110 kDa, and (3) we evaluated the relationship between the molecular weight of the MR tracers and the *R_1_* of the urine in the exocrine system. Based on the results of these studies, we were able to determine the MWCO for the filtration conducted by the exocrine system, and, furthermore, to estimate the function of the renal tubules in the freshwater mussel, *N. douglasiae*.

## RESULTS

### The structure of the exocrine system

**Fig. 1.**
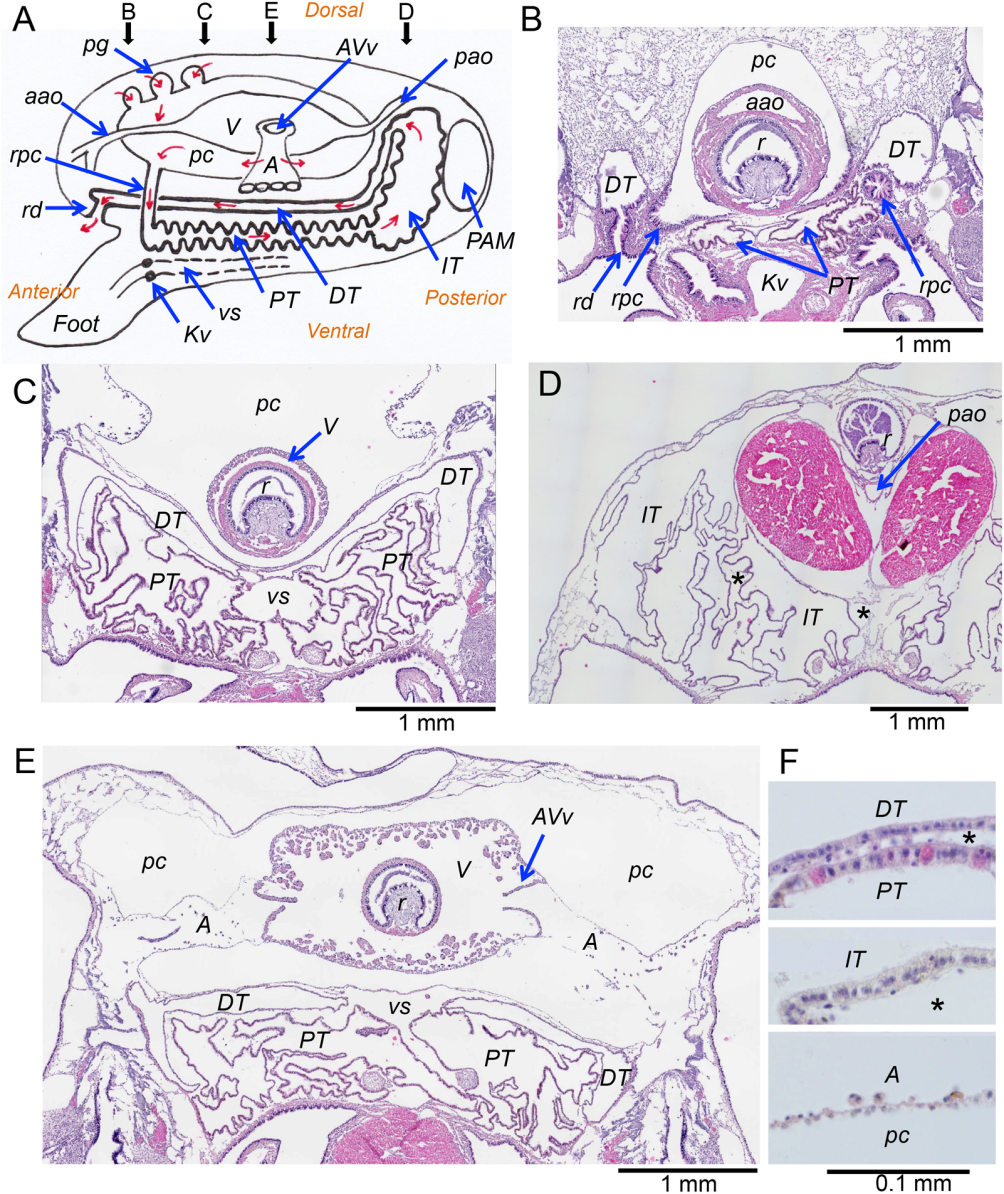
**Excretion system of *N. douglasiae.*** (A) Summary of anatomical structure. Schematic diagram of the exocrine system and adjunct organs in the longitudinal view. The direction of the urine stream is shown in red arrows. For clarity, the digestive organs and small vessels have been omitted. The position of the section is indicated by arrows labelled B to E. (B–E) Transverse section of the PFA fixed mussel (B) Section at the level of the renopericardial canal (rpc), beginning of the proximal tubule (PT), the end of the distal tubule (DT), and the renal duct (rd). (C) Section at around 1 mm anterior from the auriculoventricular valve (AVv). (D) Section at the level of the intermediate tubule (IT). (E) Section at the level of the AVv. (F) Higher magnitude images of epithelia of the DT, PT, IT and the auricle (A). Also refer to the high resolution *T_1w_-*MRI in the Movie 1. aao, anterior aorta; *Kv*, Keber's valve; PAM, posterior adductor muscle; pao, posterior aorta; pc, pericardial cavity; pg, pericardial gland; ps, pedal sinus; r, rectum; V, ventricle; vs, visceral sinus; *, interstitial space.

The exocrine system of the *N. douglasiae* was analyzed using high-resolution three-dimensional (3D) *T*_1w_-MR images and light microscopic images of Hematoxylin and Eosin (H&E) staining, and the important anatomical elements of the exocrine system were summarized in a schema of the mid-sagittal section (Fig. 1A).

The excretion system consists of the pericardial gland, auricular wall, pericardial cavity, renopericardial canal, the kidney, and the renal duct. The kidney consists of renal tubules: the proximal renal tubule (PT), the intermediate renal tubule (IT), and the distal renal tubule (DT). A pair of short renopericardial canals connected the near anterior-end of the pericardial cavity and the anterior end of PT, near Keber's valve (Fig. 1B). The PT was convoluted and distributed around the visceral sinus, running to the posterior direction (Fig. 1C). The epithelial cells were cuboidal, and had a shape similar to the brush border cell of the PT seen in vertebrates (Fig. 1F). The luminal space of the PT expanded around the posterior end of the ventricle of the heart, and thereafter, the tubule transferred to the IT. The IT filled the space between the posterior of the pericardium and the anterior of the posterior adductor muscle (Fig. 1D). Then, the IT shrank again and connected to the DT near the end of the pericardium. The DT ran to the anterior direction between the pericardial cavity and the PT (Fig. 1C). At the anterior-end of the pericardium, the DT connected with a pair of renal ducts (Fig. 1B). The epithelial cells were cuboidal, but rather flat compared with cells in the PT (Fig. 1E). Also refer to the high resolution *T_1w_-*MRI in the Movie 1.

The pericardial glands and the auricular wall are considered to be filtration sites for the hemolymph (Martin and Harrison, 1966; Bayne, 1976). As shown in Fig. 1E, the auricular wall was thin, and consists of a single flat cell layer. The pericardial glands were found around the anterior end of the pericardium, with epithelial cells similar to those of the auricular wall. Thus, the structure of the exocrine system of the *Nodularia* freshwater mussel was different from that found in the seawater mussel *Mytilus* (Bayne, 1976; Wakashin et al., 2018).

### Distribution and accumulation of the MR tracers in the kidney

**Fig. 2.**
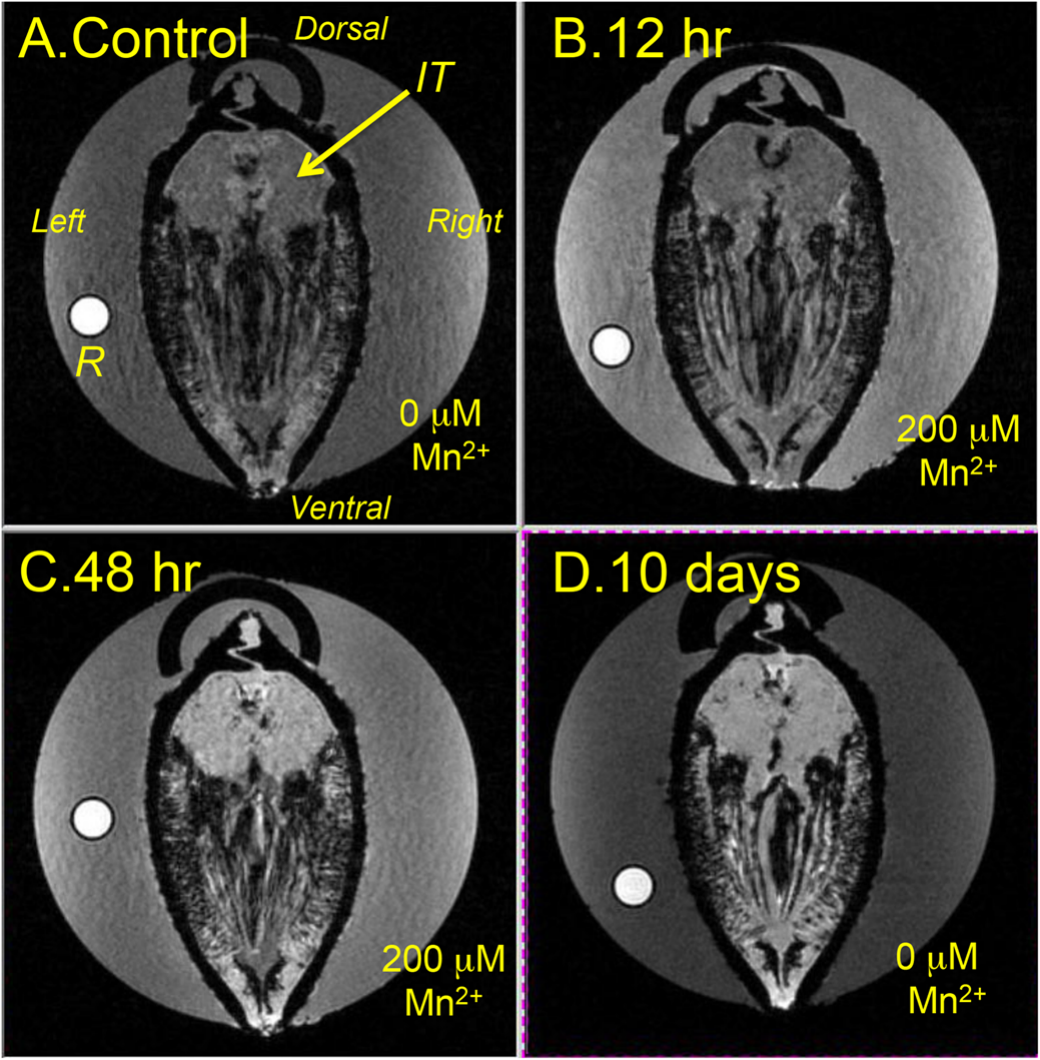
**Manganese uptake of the kidneys of *N. douglasiae.*** (A) Transverse *T*_1w_-MR image of a living mussel at the posterior side of the kidney corresponding to the IT before the addition of manganese ion. (B) Transverse *T*_1w_-MR image after 12 h (B), 48 h (C) and 10 days (D) of incubation in a pure water containing 200 µM Mn^2+^ at 20°C. Image intensity was measured compared with that of reference capillary (R) containing 0.5 mM of MnCl_2_.

Before injection study of MR tracers, we did some preliminary experiments. The *N. douglasiae* were incubated in 1 mM GdDTPA containing water for 24 h. The digestive tract was visualized in higher intensity by *T*_1w_-MRI, but the image intensity of the kidney remained the same level. Therefore, MR tracers with higher molecular weight were not uptake from the digestive tracts. We also could not detect accumulation of Mn^2+^ in the kidney of the *N. douglasiae* after 24 h incubation with 50 µM Mn^2+^. When the mussel was incubated in water containing 200 µM Mn^2+^, the *T*_1w_-MRI kidney intensities increased to 130% at 12 h, and 200% at 48 h, compared with the control, and were maintained at the same level thereafter for 10 days (Fig. 2). In the digestive tract visualized at the higher signal intensity, Mn^2+^ might be absorbed slowly from the digestive tract. Since the intensity of the 200 µM Mn^2+^ water is 180% compared with that of pure water, the Mn^2+^ concentration in the kidney could be estimated at around 200 µM. In addition, intensities in the foot also increased in a similar fashion, up to 190%. Therefore, the Mn^2+^ concentration in the kidney might be of the same order as that in the foot, and the kidney did not concentrate the Mn^2+^. Judging from these results, the function of the exocrine system in a freshwater mussel may be different from that in the seawater mussel, which can concentrate Mn^2+^ in the kidney (Wakashin et al., 2018).

**Fig. 3.**
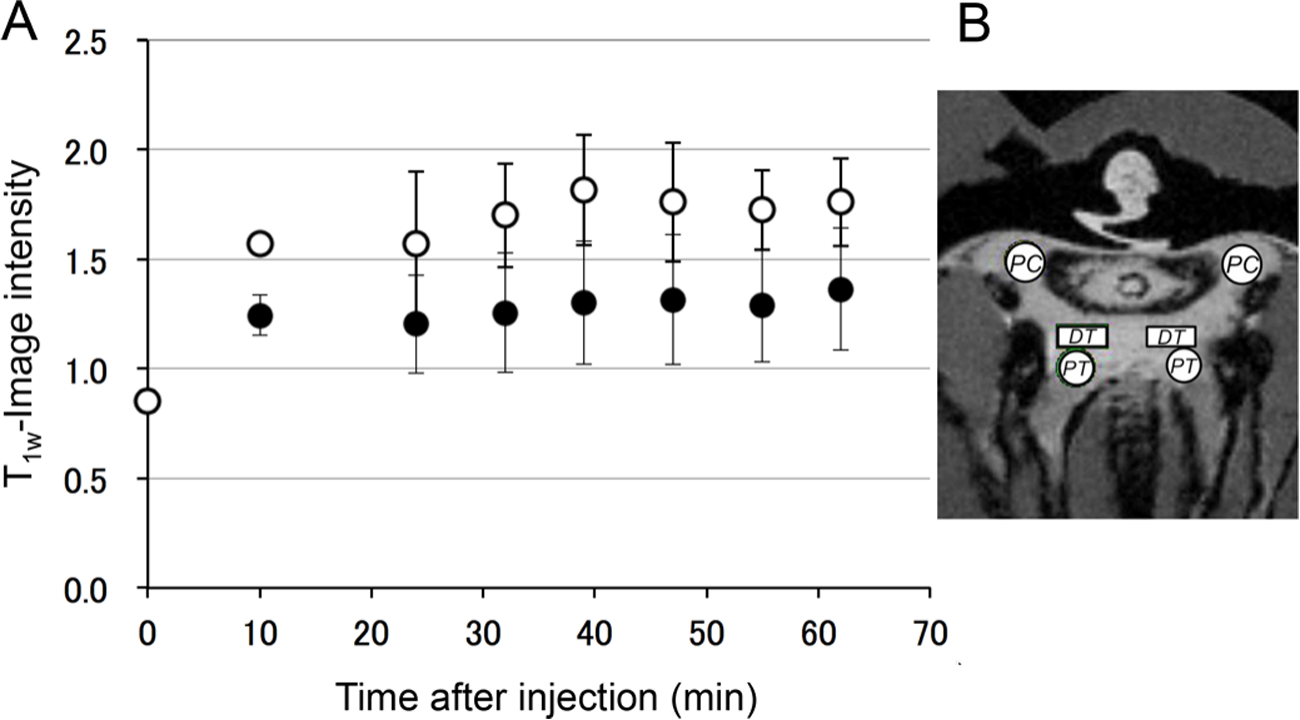
**Time course dependent changes T_1w_-MR image intensities in the excretion system of *N. douglasiae* after injection of MR tracers.** (A) Typical results obtained from a single subject in CH3-DTPA-Gd or GdDTPA. Open circles indicate the mean and s.d. of the image intensity of six ROIs of renal tubules and the pericardial cavity after injection 0.2 ml of 2 mM CH3-DTPA-Gd. Closed circles indicate the mean and s.d. of the image intensity of renal tubules and the pericardial cavity of mussel after the injection 0.2 ml of 2 mM GdDTPA. (B) Position of ROIs: the PT, the DT, and pericardial cavity (PC). A statistical difference was demonstrated when comparing the intensities obtained before and after the injection of the MR traces (*P*<0.01). However, using one-way ANOVA, no statistical differences were shown in the means from 10 to 60 min after the injection of the MR tracers (*P*>0.05).

When MR tracers were injected into the foot, the *T*_1w_-MR image intensity of the visceral increased due to the increase in *R*_1_ caused by the MR tracers. Therefore, the image intensity of the kidney was depicted as a higher signal intensity at 10 min after the injection of GdDTPA or CH3-DTPA-Gd (*P*<0.05) (Fig. 3). Then, the image intensity of the kidney remained almost constant for 1 h (*P*>0.05). Therefore, GdDTPA and CH3-DTPA-Gd were filtrated into the kidney from the hemolymph. Neither the concentration nor excretion of the MR tracers were significant in this time scale.

**Fig. 4.**
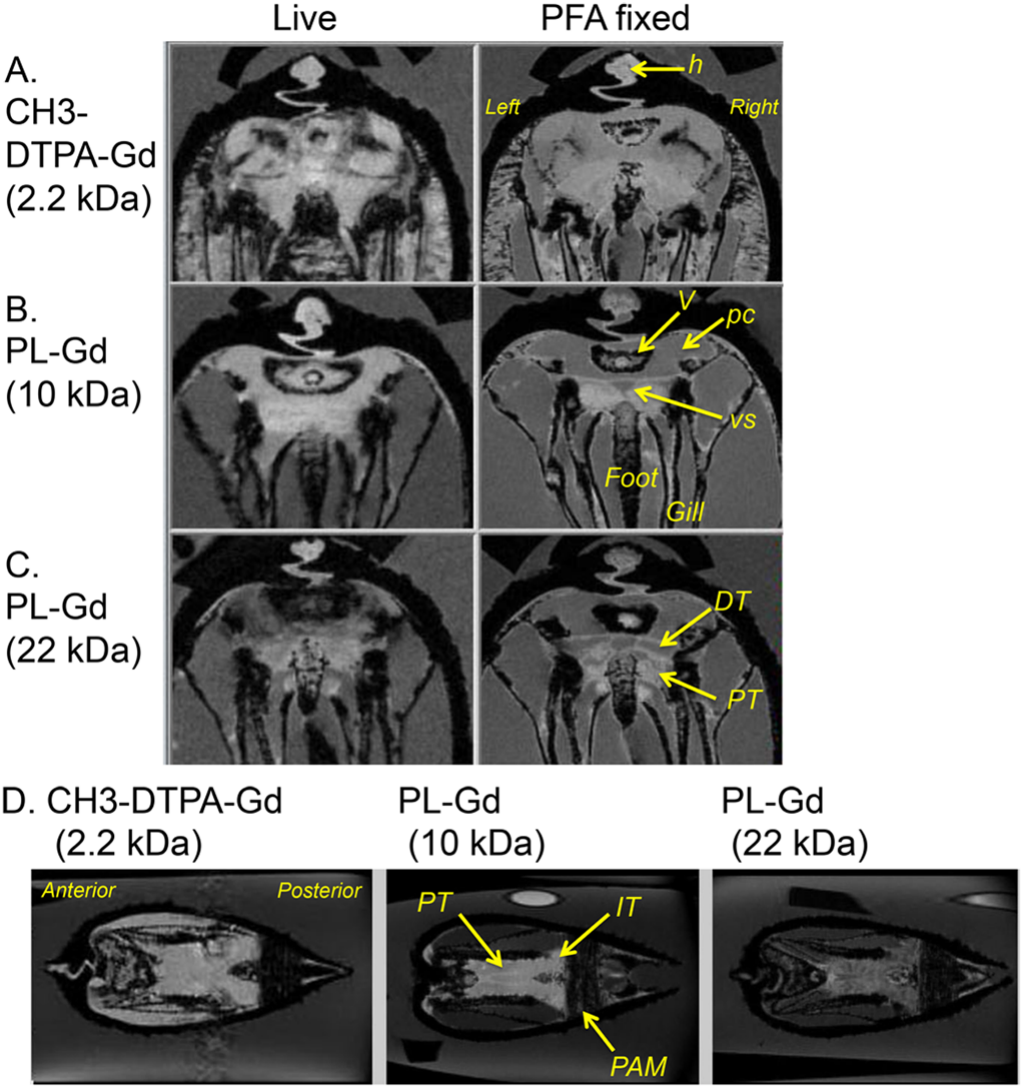
**Distribution of injected MR tracers in *N. douglasiae* imaged by *T*_1w_-MRI.** (A–C) The left column shows a transverse image at 1 mm anterior from the atrioventricular valve after the injection of (A) CH3-DTPA, (B) 10 kDa PL-Gd, and (C) 22 kDa PL-Gd. Regions of higher signal intensity compared with the water indicate the presence of the MR tracers. The right column shows a corresponding high resolution *T_1w_-*MRI of the PFA fixed mussel. (D) Coronal images at the level of the PT after injection of the MR tracers. DT, the distal tubule; h, hinge; IT, the intermediate tubule; PAM, posterior adductor muscle; pc, pericardial cavity; PT, the proximal tubule; V, ventricle of the heart; vs, the visceral sinus.

The *T*_1w_-MRI intensity of the pericardial cavity and renal tubules increased with injections of CH3-DTPA-Gd and Gd-PL 10 kDa, while injections of Gd-PL 22 kDa and 110 kDa showed only minimal or no increase. Typical transverse *T*_1w_-MR images obtained at 1 mm anterior from the auriculoventricular valve are shown in Fig. 4. As shown in Fig. 4A and B, the *T*_1w_-MR image intensities in the area of the hinge were almost the same degree compared with the PC, PT and DT, representing filtration of CH3-DTPA-Gd and PL-Gd 10 kDa in the exocrine system of the mussel. The *T*_1w_-MR image intensity of the PC, PT and DT were smaller than those of the hinge. These results indicate that the MR tracers larger than 22 kDa were not filtered in the exocrine system. Coronal images at the level along the PT to the IT are shown in Fig. 4D. These images showed almost the same *T*_1w_-MR image intensity from the initial part of the PT to the IT, indicating that the MR tracers were not concentrated along the PT.

### *R*_1_ relaxation rate of the pericardial cavity and renal tubules

We examined the difference of *R*_1_ in the exocrine system after the injection of the MR tracers. These results were summarized in Table 1. Statistically significant differences were detected in the mussel injected with CH3-DTPA-Gd by one-way analysis of variance (ANOVA), and a statistical difference was detected between the PC and the PT by Bonferroni post hoc test (*P*<0.05). We did not detect any other significant differences for the rest of the MR tracers. A small difference was also detected in the control between the PC and the PT (*P*<0.05). However, these differences were much smaller than the difference with and without the MR tracers. Thus, thereafter we used average *R*_1_ values of the PC, PT and DT for analyzing the effects of the MR tracers.

**Table 1.**
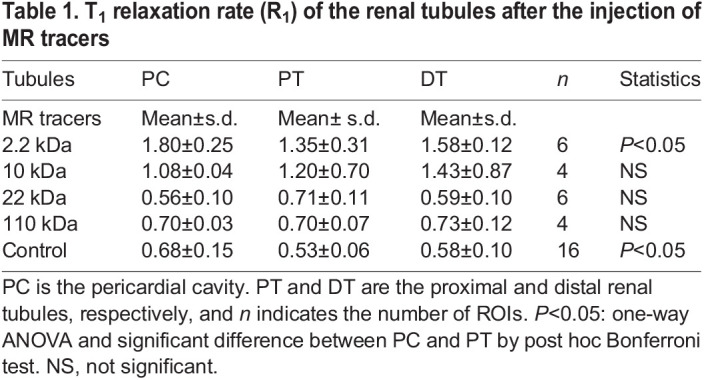
T_1_ relaxation rate (R_1_) of the renal tubules after the injection of MR tracers

**Fig. 5.**
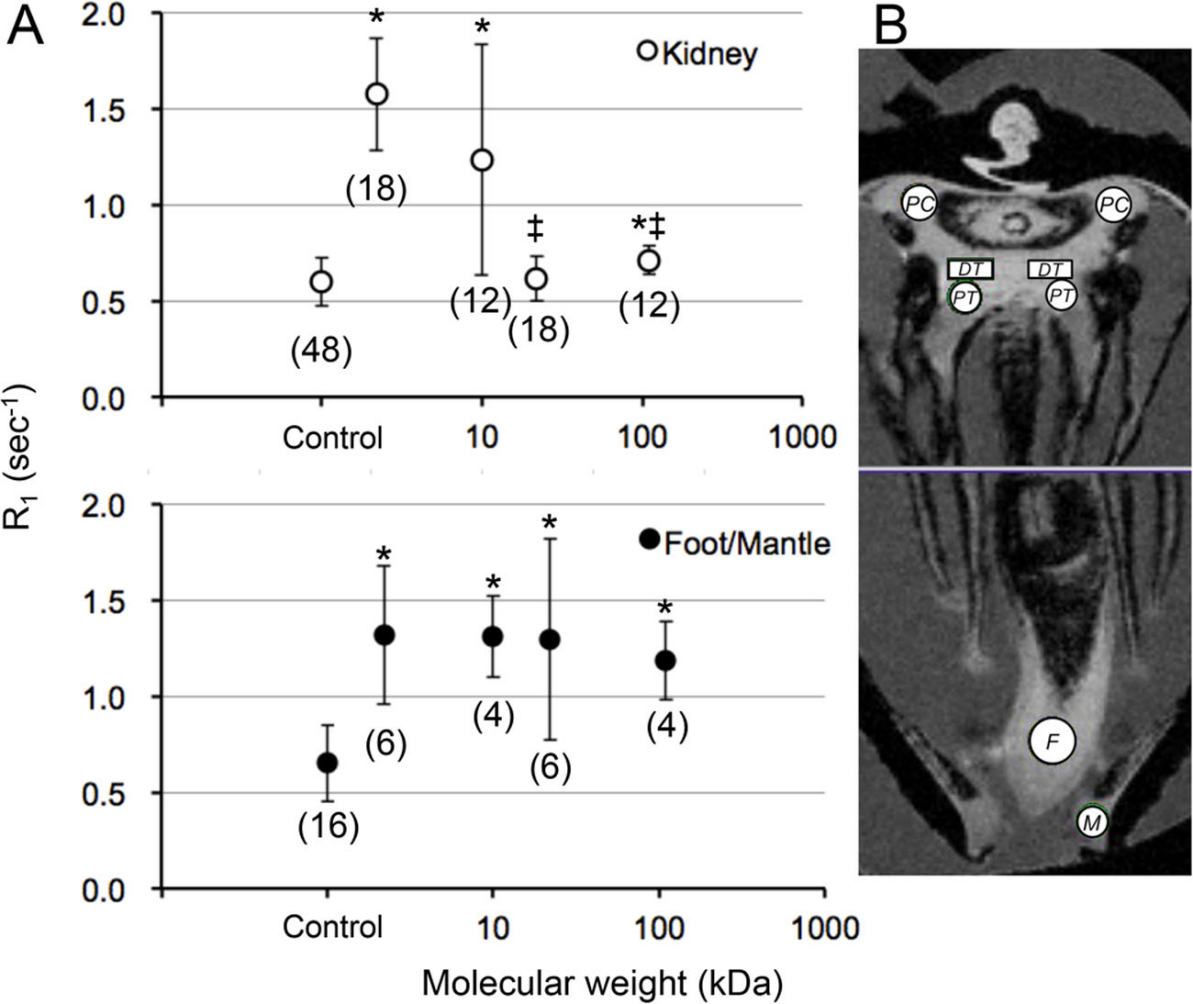
**Changes in the *T*_1_*R*_1_ of the kidney and foot/mantle after the injection of MR tracers.** (A) Means (±s.d.) of the *R*_1_ of the kidney (ROIs of the PC, PT and DT) and the visceral space (ROIs of the foot and the mantle) after the injection of the MR tracers. The number of subjects used for CH3-DTPA, 10 kDa PL-Gd, 22 kDa PL-Gd and 110 kDa PL-Gd were 3, 2, 3, and 2, respectively. The numbers of ROIs employed for the kidneys and visceral tissue studied were shown in parentheses. *, significant statistical differences compared with *R*_1_ of the control mussel (*P*<0.05). ‡, significant differences compared with *R*_1_ of the visceral tissues (*P*<0.05). (B) Position of ROIs: the PT, the DT, PC, the foot (F), and the mantle (M).

The means and s.d. of the *R*_1_ values after the injection of the MR tracers are summarized in Fig. 5. The *R*_1_ of the foot and mantle increased significantly after injection of the MR tracers, compared with the control (*P*<0.05), but there was no statistical difference shown between the *R*_1_ values of the 4 MR tracers. The *R*_1_ of the kidney (means of the PC, PT and DT) increased significantly after injection of CH3-DTPA-Gd and 10 kDa Gd-PL, compared with the control (*P*<0.05), but there were no statistical differences shown after the injection of 22 kDa and 110 kDa Gd-PL (*P*>0.05). The *R*_1_ values of the kidney were significantly smaller than those of the foot and mantle after the injection of 22 kDa and 110 kDa Gd-PL (*P*<0.05).

The concentrations of the MR tracers were estimated by C=(*R*_1_–*R*_c_)/*K*, where *R*_c_ and *K* are the *R*_1_ of the control and the relaxivity value of the MR tracers. Since the increase of *R*_1_ was around 0.8 s^−1^ and the *K* of the CH3-DTPA-Gd [4.8 (s mM)^−1^], the concentration of CH3-DTPA-Gd was about 170 µM. From a linear regression of the dose dependency of the CH3-DTPA-Gd injection, a slope of 0.490±0.026 (s mM)^−1^ (coefficient±s.e.m.) was obtained, which was 1/10 of the relaxivity of the CH3-DTPA-Gd [4.8 (s mM)^−1^]. Therefore, the injected tracers (0.2 ml) were diluted tenfold in the mussel. Since CH3-DTPA-Gd could not enter cells, the total volume of the hemolymph could be estimated as 2.0 (1.8–2.2) ml (95% confidence limits).

## DISCUSSION

### Molecular size dependency for filtration of exocrine system

The MR tracers GdDTPA (0.55 kDa), CH3-DTPA-Gd (2.2 kDa) and 10 kDa Gd-PL (10 kDa) appeared in the PC and renal tubules, but 22 kDa Gd-PL (22 kDa) and 110 kDa Gd-PL (110 kDa) did not appear. From these results we concluded that the MWCO for filtration is around 22 kDa, which means it is possible to filtrate inulin (5.5 kDa). Inulin is the common substance for measurement of the glomerular filtration rate (Gaudino and Levitt, 1949). Therefore, the exocrine system of the *N. douglasiae* might have a similar or a larger MWCO, compared with the *Anodonta Cygnea*, which lives in freshwater (Potts, 1954), and the MWCO is 50 times larger than that of the seawater mussel, *M. galloprovincialis* (Wakashin et al., 2019). Filtration of inulin was reported in the abalone and the octopus, which live in seawater, and the giant African snail, which lives on land (Martin and Harrison, 1966). Therefore, the large MWCO is not likely to be a specific function of the exocrine system of the Mollusca, which lives in freshwater.

### Function of the counter-current system of renal tubules

**Fig. 6:**
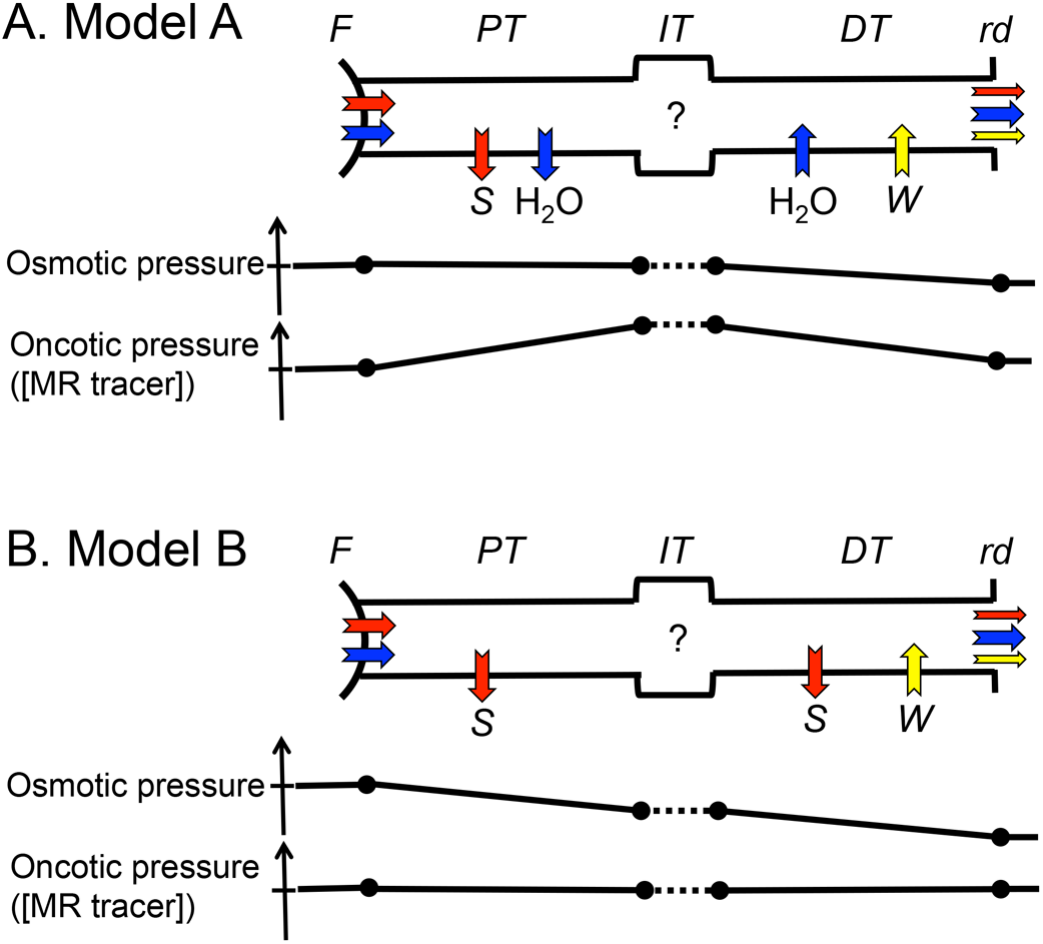
**Schematic diagrams of absorption and secretion of solutes and water along the renal tubules.** (A) Model A: (1) solutes smaller than 20 kDa (red arrow) and water (blue arrow) are filtered in the auricular wall and pericardial gland (F). (2) The PT reabsorbs small useful solutes (S) such as Na^+^ and amino acids with reabsorption of water (H_2_O) by isotonic osmosis. (3) The DT excretes water by the oncotic pressure caused by the non-absorbed solutes, and may actively secret waste molecules (W). Changes in the osmotic pressure and oncotic pressure of luminal fluid are presented as graphs. In this system, the osmotic pressure does not change, and the apparent concentration of non-absorbed solutes, such as the MR tracers ([MR tracer]), should be increased in the PT and should be decreased in the DT. (B) Model B: (1) solutes smaller than 20 kDa and water are filtered in the auricular wall and pericardial gland. (2) The PT tubule reabsorbs solutes (S) without reabsorption of water, (3) The DT tubule reabsorbs electrolytes (S) and may actively secret waste molecules (W) without any transport of water. In this system, the osmotic pressure of the luminal fluid in the PT should be decreased by reabsorption of solutes, while the apparent concentration of the MR tracers is constant. Functions in the IT remain an open question (?) in both hypotheses.

As concerned water balance, the kidney in seawater mussels conserve water against the hyperosmotic seawater. Therefore, the kidney of the *Mytilus* has a smaller MWCO (0.5 kDa) and can concentrate urine so that reabsorb water to maintain a certain level of body water (Wakashin, et al., 2018, 2019). On the contrary, the freshwater mussel, such as *N. douglasiae*, has the risk of dilution of body water due to the hypoosmotic freshwater. If filtrated fluid is excreted as it is, the mussel will lose useful substances, such as electrolytes and glucose. Therefore, the exocrine system has to reabsorb useful substances without reabsorbing water, and has to excrete any excess amount of water to prevent dilution of the body fluid. Based on our histological investigation, the renal tubules of *N. douglasiae* contain a counter-current system. A similar counter-current system was found in the kidney of the *Anodonta* (Andrews and Jennings, 1993). Andrews commented that “the proximal limb of the kidneys are released for the resorption of ions, excretory products being added in the distal limb” (Andrews, 1988). Indeed, the epithelial cells of the PT had a shape similar to that of the brush border cell of the proximal tubule found in vertebrates, and the epithelial cells of the DT had shape similar to the cell of the collecting duct of vertebrates (Fig. 1). The slow change of the MR tracers in the exocrine system (Fig. 3) suggests a low filtration rate, and this is in good agreement with the low filtration rate of the *Anodonta* (0.01 ml/hr/g wet weight) estimated by inulin clearance (Potts, 1954). The low filtration rate also allows for the time required to uptake useful solutes along the renal tubules. We have postulated two systems, as follows (Fig. 6).

Model A: (1) the exocrine system filtered out molecules smaller than 20 kDa, (2), so the PT tubule can reabsorb small useful solutes, such as Na^+^ and amino acids with reabsorption of water via isotonic osmosis, and the concentration of the non-absorbed solutes increased in the tubule, (3) and the DT excretes water due to the oncotic pressure caused by the non-absorbed solutes, and the DT may actively secret waste molecules. This is similar to the reabsorption found in the proximal tubules of the vertebrate (Guyton and Hall, 2006). Since luminal fluid in the PT remains in the isosmotic, reabsorption of solutes works the most efficiently via osmosis. In this system, the apparent concentration of non-absorbed solutes, such as the MR tracers, should be increased in the PT, and should be decreased in the DT (Fig. 6A).

Model B: (1) the exocrine system filters out molecules smaller than 20 kDa, (2) the PT tubule reabsorbs small useful solutes without the reabsorption of water, (3) and the DT tubule reabsorbs electrolytes and may actively secret waste molecules without any transport of water. This is similar to the counter-current system seen in the vertebrate without vasopressin or producing diluted urine (Guyton and Hall, 2006). In this system, the osmotic pressure of luminal fluid in the PT should be decreased by the reabsorption of solutes, while the apparent concentration of the MR tracers should remain constant (Fig. 6B). The long pathway of the IT and PT might be beneficial for additional reabsorption and/or secretion (Andrews, 1988).

As shown in our results for MR tracers smaller than the MWCO in Table 1, the *R*_1_ values of the PT and DT were not higher than that of the PC. As explained in the Introduction, the *R*_1_ value depends concentration of MR tracers: *R*_1_=*R*_0_+K·C, where *R*_0_, K and C are the intrinsic *R*_1_ of the hemolymph, the relaxivity value of the MR tracer (mM^−1^ s^−1^), and the concentration of the MR tracer (mM), respectively. Therefore, no increase of *R_1_* values along the renal tubules indicates no increase of concentration of MR tracers in the urine. Thus, there was no water reabsorption in the renal tubules. In the *Anodonta* kidney, urine was hypotonic by 40% of the salt that was reabsorbed by the kidney (Picken, 1937; Martin and Harrison, 1966). The luminal fluid in the PT might be hypoosmotic at around 20 mosmol kg^−1^ compared with the hemolymph in Unionoidae (40–50 mosmol kg^−1^) (Picken, 1937; Burton, 1983; Potts, 1954; Byrne and Dietz, 1997). In addition, even with a small difference, as in the case with CH3-DTPA-Gd (2.2 kDa), the *R*_1_ of the PT was smaller than that of the PC (*P*<0.05). If this is true, the decrease of *R*_1_ indicates secretion of water in the PT, because larger molecules such as polypeptides and immunoglobulins are reabsorbed by endocytosis (Boron and Boulpaep, 2004) and it is not likely that the mussel has a specific transporter for CH3-DTPA-Gd. We could not estimate a specific transport system of water. However, the active transport system using ATP can generate a gradient of 100 mosmol kg^−1^ (Guyton and Hall, 2006), and that could overcome the osmotic gradient caused by even the total osmotic concentration of hemolymph (40–50 mosmol kg^−1^). The oncotic pressure of the luminal fluid caused by large molecules may also prevent water reabsorption through the epithelia of the renal tubule. Judging by these results, we doubt that model A is the actual case, and feel inclined to believe that model B is actually the true case, or at least close, for transport in the counter-current system of the *N. douglasiae*. Roles of the IT remains an open question.

In conclusion, we (1) injected five MR tracers in *N. douglasiae*, and detected the accumulation of the MR tracers in the exocrine system by MRI, and (2) MR tracers smaller than 10 kDa appeared in the exocrine system, but MR tracers larger than 22 kDa did not. Thus, the MWCO is around 22 kDa. (3) MR tracers were not concentrated in the renal tubules, and (4) the renal tubules consist of a counter-current system, which suggested the reabsorption of useful molecules and the excretion of water to prevent any dilution of hemolymph. (5) A main renal function in the freshwater mussel is the excretion of water, which is opposite to that of the seawater mussel and the vertebrate, where one main function is to preserve water.

## MATERIALS AND METHODS

### Experimental mussels

*Nodularia douglasiae* (Gray, 1833) were supplied by Sato Craft (Okayama, Japan). These mussels were collected from a river in Seki, Okayama in June of 2016 and December of 2019. The species of the mussels was identified by sequencing of the mitochondrial cytochrome c oxidase subunit I gene (Folmer et al., 1994; Seo and Seo, 2019). After collection, the mussels were immersed in water, and then transported to the laboratory by a refrigerated transport service, and maintained at 10°C, within a time period of 16 h (Cool Ta-Q-BIN, Yamato Transport Co., Ltd, Tokyo, Japan). At the laboratory, the mussels were housed in aerated water (4 l) with glass beads (0.3–1.0 mm diameter; 5 cm depth) in a 6 l bath at room temperature (20–25°C). The mussels were fed with *Chaetoceros calcitrans* (WDB Environmental & Biological Research Institute, Tokushima, Japan), and 2.5×10^6^ cells/l water in the bath were applied at intervals of 3–4 days. A total of 21 mussels were used in this study. The length, height and width of the mussels were 38.4±2.2 mm, 20.7±1.2 mm, 15.2±1.4 mm (means and s.d.), respectively. All of the animal experiments in this study were carried out under the rules and regulations of the ‘Guiding Principles for the Care and Use of Animals’ set by the Physiological Society of Japan, and approved by the Animal Research Councils at Dokkyo University School of Medicine.

### MR tracers and anesthetic chemicals

Five kinds of MR tracers were employed in the tracer study as follows: GdDTPA (Magnevist, Schering, Berlin, Germany; 0.55 kDa), CH3-DTPA-Gd (NMS-60, Nihon Medi-Physics, Chiba, Japan; 2.2 kDa), and Gd-PL (Gd-DTPA-polylysine 10 kDa, 22 kDa and 110 kDa, BioPAL, Worcester, MA, USA). Two mM Gd-DTPA and CH3-DTPA-Gd dissolved in 20 mM NaCl solution (Burton, 1983), and Gd-PLs solution containing 10 mg polylysine/ml were used for the injections to the mussels.

Tricaine was used as an anesthetic (Lellis et al., 2000). Equivalent volumes of 0.1 g/ml tricaine solution (Tricaine methanesulfonate, FUJIFILM Wako Pure Chemical, Osaka, Japan) and 0.046 g/ml tris buffer solution (Trizma base, Sigma-Aldrich, St Louis, MO, USA) were mixed into water at the final concentration of 500 ppm or 750 ppm, and at a pH of around 7.

The mussels were sedated by 250 ppm and anesthetized by 500 or 750 ppm tricaine (Lellis et al., 2000), followed by injections of 0.2 or 0.1 ml of the MR tracers into the foot using a 30 G needle (Nipro, Osaka, Japan). Thereafter the mussels were incubated in pure water. The MR tracer injections were confirmed by noting a higher signal intensity of the visceral mass in scout *T*_1w_-MR images those are a set of images to identify the relative anatomical position of a collection of MR images.

### Magnetic resonance imaging

The MRI examination of the *N. douglasiae* in this study used procedures noted in our previous report (Seo and Seo, 2019). In brief, the mussels were placed in a plastic tube (inner diameter of 22.5 mm), and were immersed in 12 ml of water without aeration, and the temperature was kept at 20°C by variable temperature control units (ECU-20 and BVT-2000, Bruker Biospin, Ettlingen, Germany). Water was exchangeable through another tube set in the bottom of the tube holding the mussel. The ^1^H MR images were obtained by a 7 T vertical MR microimaging system (AVANCE III, Bruker Biospin, Ettlingen, Germany) with ParaVision operating software (version 5.1) and equipped with an active shielded gradient (micro2.5) and a 25-mm ^1^H birdcage radiofrequency coil.

*R*_1_ relaxation rate was measured by a two-dimensional saturation-recovery imaging (2D SR T_1_-MRI) method with five relaxation delays from 0.1 s to 4 s. The pixel size was 180×180 µm and the thickness of slice was 1 mm. The total image acquisition time was 9 min 21 s. The *R*_1_ image was calculated by the Image Sequence Analysis Tool provided by ParaVision. Images with a large motion artifact were omitted from the following data analysis. In order to include the whole-body structure of the mussel, 3D *T*_1_-weighted gradient-echo imaging (*T*_1w_-MRI) was used. The MR signal in the kidney was analyzed by 3D *T*_1w_-MRI with a voxel size of 240×240×240 µm, and a combination of *T*_R_/*T*_E_/θ=50 ms/2.5 ms/45°, where *T*_R_, *T*_E_ and θ indicate repetition time, echo-time and flip angle, respectively. The total image acquisition time was 7 min 40 s. Images were Fourier-transformed with a data matrix of 256×128×128 after zero-filling of data, and the final voxel size was 180×180×180 µm. For a higher image resolution, 3D slab *T*_1_-weighted gradient-echo imaging was employed with a voxel size 90×90×90 µm with a combination of *T*_R_/*T*_E_/θ=50 ms/3.2 ms/45°. Motion artifacts of the mussel were reduced by 500 ppm tricaine. The image intensity of the *T*_1w_-MR image was quantified by comparison with the image intensity of a reference capillary containing 0.5 mM MnCl_2_ solution. Statistical analysis of *R*_1_ was applied using the unequal variance *t*-test (Welch's test) or one-way ANOVA employing Excel 2016 (Microsoft, Redmond, WA, USA). *P*-values less than 0.05 were regarded as significant.

### Anatomical structure

Anatomical information for each 4% paraformaldehyde (PFA) fixed mussel was obtained by 3D *T_1w_-*MRI, with a voxel size of 60×60×60 µm, and a combination of T_R_/T_E_/θ=50 ms/3.75 ms/45°. For the histological studies, the paraffin sections of the PFA fixed mussel were prepared using a slice thickness of 10 µm. The sections were stained by H&E staining. Images were detected by microscopes (BZ-X710, Keyence, Osaka, Japan; BX63, Olympus, Tokyo, Japan) with an image-stitching mode that is the process of combining multiple images with overlapping fields of view to produce a wide field of image.

### Protocols employed in the MR experiments

Experiments on the accumulation of the MR tracers in the kidney started from the low-resolution 3D *T*_1w_-MRI (7 min 40 s) before injection of the MR tracers. Then, mussels were anesthetized by tricaine, and thereafter, we waited for 30–60 min until the foot extended. After injection of MR tracers into the foot, the mussel was returned to the pure water and the low-resolution 3D *T*_1w_-MRI were measured for 1 h.

Experiments on filtration of the MR tracers started from measurement of the low-resolution 3D *T*_1w_-MRI (7 min 40 s) and a pair of 2D SR T_1_-MRI (18 min 40 s) before the injection of the MR tracers. Then, the mussels were anesthetized by tricaine, and incubated for 30–60 min until the foot extended. After injection of the MR tracers into the foot, the mussel was returned to the pure water for 15 min. The low-resolution 3D *T*_1w_-MRI was measured to confirm uniform distribution of the MR tracers. Then, a pair of 2D SR T_1_-MRI were measured at around 45 min after the injection. When necessary, the high-resolution 3D *T*_1w_-MRI was measured thereafter under the anesthesia. The *R*_1_ value obtained in separate experiments were included as the *R*_1_ for the control condition.

## Supplementary Material

Supplementary information
