# Supplementary material for: The kidney of the Nodularia freshwater mussel has a larger filtration-size and counter-current system with improved water excretion compared with the seawater mussel Mytilus
Source: Biol Open. 2021 Jun 8;10(6):bio058692. doi: 10.1242/bio.058692 (PMC8214426; doi:10.1242/bio.058692)
Supplement: Supplementary information [file biolopen-10-058692-s1.pdf]

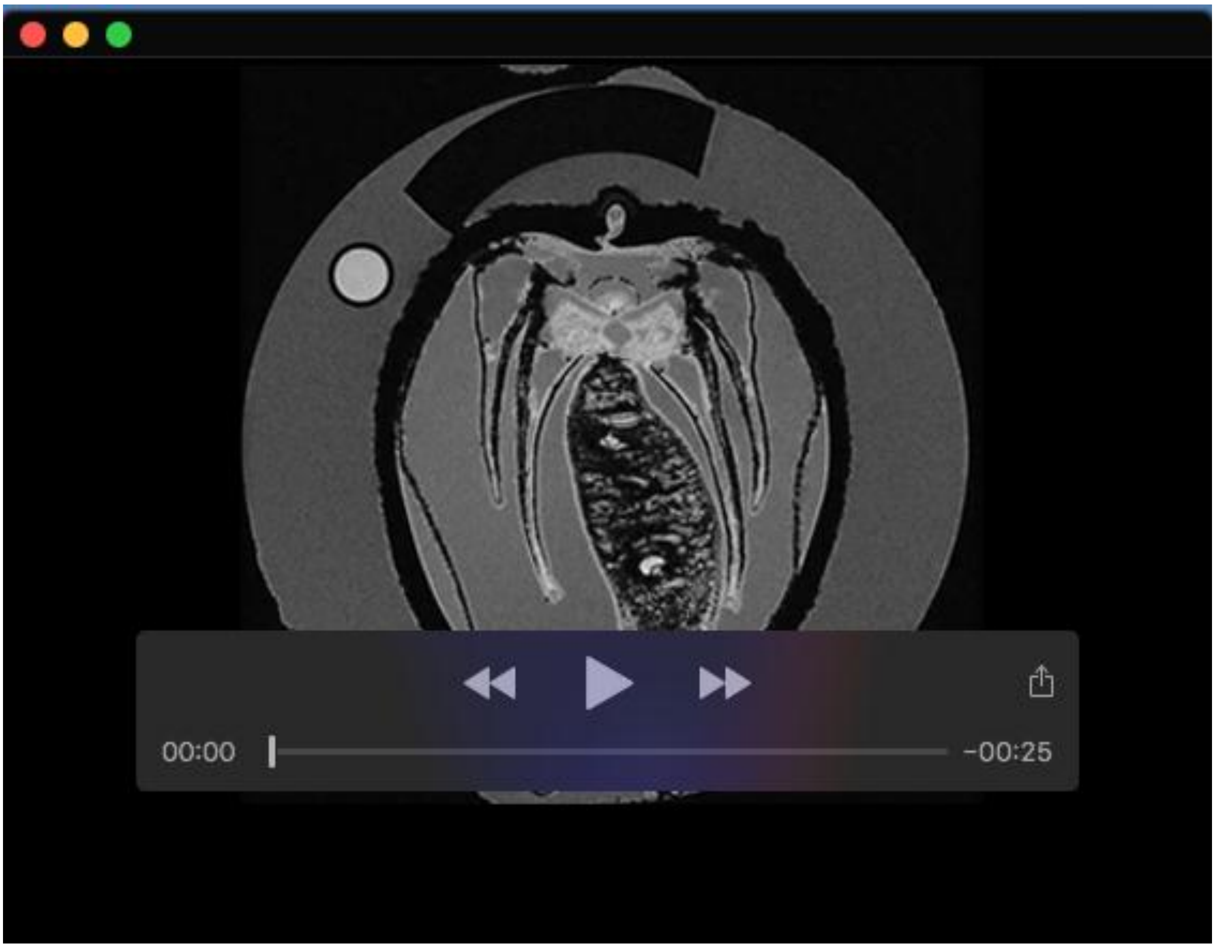

Movie S1. Transverse T_{1w}-MR images of the renal system of PFA fixed *Nodularia douglasiae*. Field of view is 23 x 23 x 13.4 mm that covers from the anterior aorta to the posterior adductor muscle shown in the schema of Fig. 1A. The voxel resolution is 45 µm. One image is shown in Fig. 4C.

Biology Open • Supplementary information
